# Characterization of microsatellite markers developed from *Prosopis rubriflora* and *Prosopis ruscifolia* (Leguminosae - Mimosoideae), legume species that are used as models for genetic diversity studies in Chaquenian areas under anthropization in South America

**DOI:** 10.1186/1756-0500-7-375

**Published:** 2014-06-18

**Authors:** Fábio M Alves, Maria I Zucchi, Ana MG Azevedo-Tozzi, Ângela LB Sartori, Anete P Souza

**Affiliations:** 1 Departamento de Biologia Vegetal (BV), Instituto de Biologia, Universidade Estadual de Campinas - UNICAMP, Cidade Universitária Zeferino Vaz, CP 6109, CEP 13083-970 Campinas, SP, Brazil; 2 Centro de Biologia Molecular e Engenharia Genética (CBMEG) - Universidade Estadual de Campinas - UNICAMP, Cidade Universitária Zeferino Vaz, CP 6010, CEP 13083-970 Campinas, SP, Brazil; 3 Pólo Apta Centro Sul, Rod. SP 127 km 30, Piracicaba, SP CP 28, CEP 13400-970, Brazil; 4 Departamento de Biologia, Centro de Ciências Biológicas e da Saúde (CCBS), Universidade Federal de Mato Grosso do Sul – UFMS, Cidade Universitária s/n, Caixa Postal 549, CEP 79070-900 Campo Grande, MS, Brazil

**Keywords:** *Prosopis*, Pantanal, Chaco, Population Genetics, Conservation, Short Tandem Repeats

## Abstract

**Background:**

*Prosopis rubriflora* and *Prosopis ruscifolia* are important species in the Chaquenian regions of Brazil. Because of the restriction and frequency of their physiognomy, they are excellent models for conservation genetics studies. The use of microsatellite markers (Simple Sequence Repeats, SSRs) has become increasingly important in recent years and has proven to be a powerful tool for both ecological and molecular studies.

**Findings:**

In this study, we present the development and characterization of 10 new markers for *P. rubriflora* and 13 new markers for *P. ruscifolia*. The genotyping was performed using 40 *P. rubriflora* samples and 48 *P. ruscifolia* samples from the Chaquenian remnants in Brazil. The polymorphism information content (PIC) of the *P. rubriflora* markers ranged from 0.073 to 0.791, and no null alleles or deviation from Hardy-Weinberg equilibrium (HW) were detected. The PIC values for the *P. ruscifolia* markers ranged from 0.289 to 0.883, but a departure from HW and null alleles were detected for certain loci; however, this departure may have resulted from anthropic activities, such as the presence of livestock, which is very common in the remnant areas.

**Conclusions:**

In this study, we describe novel SSR polymorphic markers that may be helpful in future genetic studies of *P. rubriflora* and *P. ruscifolia*.

## Findings

### Background

The genus *Prosopis* L. belongs to the Leguminosae botanical family, which contains 44 species. *Prosopis* L. is predominantly restricted to the neotropics [1]. *Prosopis rubriflora*[2] and *Prosopis ruscifolia*[3] are tree species known locally as “espinheiro” and “algarroba,” respectively. These species are important both economically and ecologically. For example, the fruits and seeds of *P. ruscifolia* are reported to be good sources of nutrition for humans and animals [4], and the flowers of *P. rubriflora*, which are present throughout the year, provide important food resources, such as pollen and nectar, for the local fauna [5]. *P. rubriflora* has a narrow distribution range and is limited to Paraguay and Brazil, but *P. ruscifolia* is also found in Argentina and Bolivia [6,7].

In Brazil, *P. rubriflora* and *P. ruscifolia* are associated with Chaquenian areas [8] and are limited to the southern portion of the Pantanal [9,10]. Both species are excellent indicators of Chaquenian areas in Brazil; *P. rubriflora* is usually associated with arboreal physiognomy, and *P. ruscifolia* is frequently associated with forest physiognomy. Both species can be used as models for genetic studies of diversity in these areas.

While estimating genetic diversity, the use of molecular markers has been helpful in defining alleles and studying genetic flow, population structure, paternity, inheritability, genetic maps and conservation genetics [11]. Simple sequence repeat markers (SSRs), commonly referred to as microsatellite markers, are desirable tools because they are co-dominant in nature, multi-allelic and widely distributed in the genome; they are also currently cheap, reproducible and relatively easy to analyze [12]. This work reports the development, characterization and transferability of microsatellite markers for *P. rubriflora* and *P. ruscifolia*.

### Construction of a microsatellite-enriched library

DNA was extracted from *P. rubriflora* and *P. ruscifolia* using the DNeasy® Plant Mini Kit (Qiagen, Hilden, DE) according to the manufacturer’s instructions. Microsatellite-enriched libraries for *P. rubriflora* and *P. ruscifolia* were constructed as described by Billote *et al.*[13]. The genomic DNA was digested with *Afa*I after enrichment with streptavidin-coated magnetic beads (Streptavidin MagneSphere Paramagnetic Particles, Promega, Madison, WI); biotinylated (CT)_8_ and (GT)_8_ microsatellite probes were added for the dinucleotide-enriched library. The fragments were amplified by PCR and cloned into the pGEM-T vector (Promega, Madison, WI). XL1-Blue (*Escherichia coli*) competent cells were transformed with the recombinant plasmids and then cultivated on agar medium containing ampicillin (100 mg/ml), X-galactosidase 2% (100 μg/ml) and IPTG (100 mM). The selected clones were added to a Big Dye Terminator v3.1 Cycle Sequencing Kit (Applied Biosystems, Foster City, CA) and sequenced using an ABI 377 sequencer (Applied Biosystems, Foster City, CA). The sequences were aligned and edited using SeqMan Software (DNAStar, Madison, WI), and the adapters and restriction sites were removed using Microsat Software (A. M. Risterucci, CIRAD, personal communication). To identify microsatellite-enriched regions, we used the Simple Sequence Repeat Identification Tool (SSRIT) [14] and defined the following numbers of repeats/motifs: five/dinucleotides, four/trinucleotides and three/tetra- or pentanucleotides. After these steps, primers were designed using the PrimerSelect software (DNAStar, Madison, WI).

### Fragment amplification

The fragments were amplified using polymerase chain reactions containing 8 ng of template DNA, 2 mM MgCl_2_, 50 mM KCl, 20 mM Tris–HCl (pH 8.4), 0.2 mM dNTPs, 0.19 mg/ml BSA (bovine serum albumin), 0.15 mM of each primer and 1 U of *Taq* DNA polymerase; the reactions were then brought to a final volume of 20 μl with ultrapure water. To define the temperatures for the PCR reactions, we adopted the guidelines described by Mottura *et al.*[15]; for the annealing temperatures, we used a gradient program with temperatures ranging from 65°C to 55°C. The samples were collected in the Chaco remnants of Corumbá and Porto Murtinho, Mato Grosso do Sul, Brazil. Twenty *P. rubriflora* samples were collected in each of two Chaco remnant locations: Fazenda São Manoel (FSM) (21°47′44.5″S; 57°39′34.6″W) and Fazenda Santa Vergínia (FSV) (22°06′40.5″S; 57°49′57.6″W). Twenty-three *P. ruscifolia* samples were collected in Estação do Carandazal (ECD) (19°48′33.2″S; 57°10′11.0″W), and 25 samples were collected in Fazenda Retiro Conceição (FRC) (21°42′23.7″S; 57°45′58.2″W). The cross-amplification of the markers was evaluated in 5 *P. rubriflora* samples obtained from FRC (21°41′00.7″S; 57°46′43.8″W) and 5 *P. ruscifolia* samples from Chácara Jacaré (21°41′20.1″S; 57°52′15.5″W) using the same conditions as for the native species. The amplified samples were genotyped by vertical electrophoresis using denaturating polyacrylamide gels (6%), and DNA bands were visualized using silver nitrate [16]; the sizes of the resulting fragments were estimated by comparison to a 10-bp DNA ladder (Invitrogen, Carlsbad, CA). Statistical analyses were performed using Microsatellite Toolkit v.3.1.1 [17] to calculate the expected heterozygosity (H_e_), observed heterozygosity (H_o_) and polymorphism information content (PIC). The Genepop software v.1.2 [18] was used to estimate adherence to Hardy-Weinberg (HW) equilibrium and possible linkage disequilibrium (LD), and the frequency of null alleles was estimated using FreeNA [19].

### Results and discussion

We designed 32 primer pairs: 13 for *P. rubriflora* and 19 for *P. ruscifolia*. However, only 10 of the *P. rubriflora* primer pairs and 13 of the *P. ruscifolia* primer pairs amplified properly. The nine remaining pairs of primers were discarded because amplification errors were observed in the preliminary tests. Polymorphisms were detected in 9 of the native *P. rubriflora* markers and 12 of the native *P. ruscifolia* markers; only one marker from each species had a monomorphic pattern based on the populations analyzed. Eight markers from *P. rubriflora* successfully cross-amplified and were polymorphic for the tested samples, and 2 markers failed during cross-amplification. Eleven *P. ruscifolia* markers were successfully cross-amplified; 7 were polymorphic, and 2 failed this analysis (Table 1).

**Table 1:** **Primers developed for *Prosopis rubriflora and Prosopis ruscifolia***

**Marker**	**GenBank register no.**	**Primer sequences (5′-3′)**	**Motifs**	**T**_**a**_**(°C)**	**Size**^**a**^**(bp)**	**Crossed amplification**
Prb1	KF923365	F: AACTACCGCAGCACTTTTCAGA	(gt)7	62.7	255-267	267^b^
		R: ACTACTTGGAGATGCCGTGGA				
Prb2	KF923366	F: GAAAGCCGCGCTCCTAAG	(gc)4(ac)7	61.0	140-146	126^b^
		R: ATTCTTTTGTGTCTTGTCTTCTCG				
Prb3	KF923367	F: TCCAAAGACCGCAAGAAGAT	(ca)7	61.0	149-159	145^b^
		R: AGGCCAAAAAGGACTCAAAAT				
Prb4	KF923368	F: ATCCGATAAATACACCTTCTGG	(ca)8	61.0	194-230	203^b^
		R: GGTGTATCGTAAAAGCCTGG				
Prb5	KF923369	F: TTTAAACATTGCACGTGAACCTAT	(ac)9	56.4	149-155	-
		R: TTCACCCCTAAACCCCCTT				
Prb6	KF923370	F: TCATCTCTCAAAGAAAACGCACTC	(tg)10	56.4	115-133	125^b^
		R: CCGCAGAGAAGCCCCTACATA				
Prb7	KF923371	F: GGCTTAGCATCACCCTCCAT	(ac)8	61.0	219-225	220^b^
		R: CTTACCCTTTCAGTCCATTTACCA				
Prb8	KF923372	F: CAACACCAAAACGGCGAGATGAT	(gt)13	61.0	144-164	154^b^
		R: TTCGCCAAACGCCAGCATTAG				
Prb9	KF923373	F: TTCTTCTCCTTCTTCATCTTCCTCC	(ac)9	62.7	167-175	190^b^
		R: ACAACGTTGATCCCAAAACCTAAG				
Prb10	KF923374	F: TTTTGGTGGATTTGATAGAGCC	(tca)5	56.4	223	-
		R: GAGTGGGGTCAAGAAAGAACAG				
Prsc1	KC753210	F: AATGGAGTTTGTTTGTGTCTGTGG	(ac)9(ct)5	56.5	279-297	300^b^
		R: ATTACGGATACATCGAGCCTTCTT				
Prsc2	KC753211	F: GCGGAATTCCAAACGACAA	(ac)9	64.7	224-252	250^b^
		R: ACAGCAACACCCTCACTCTCAA				
Prsc3	KC753212	F: CCACAAGCACACGCACACTCAGAC	(ca)6	64.7	156-160	122
		R: CCAGCACTAGACTTCGCCACCAAC				
Prsc4	KC753213	F: CAAAATCCAACAAATAAACACACC	(caa)2(ga)4	63.9	218-232	230^b^
		R: GGCGGATTCTTGGCTCTCT				
Prsc5	KC753214	F: CGCGTTAAGTCTGCCTTGCTTT	(gt)8	59.0	220-240	218
		R: CTCATGGTATTTCCCTTGTCGTCC				
Prsc6	KC753215	F: CGAGCGGCGAAAAATGATAAA	(gt)8	63.9	184-210	200
		R: GCTGCTTCCCATAATCCTCTCCT				
Prsc7	KC753216	F: CAGGGATTTAATCTCTTTGGTGTAG	(tg)8…(gtgg)2(gt)5	59.0	122-156	122^b^
		R: ACAAGCTGGAAAGAGTCGCA				
Prsc8	KC753217	F: AGTGACGTGAACACGCTGAGG	(tg)10	62.7	98-120	114^b^
		R: TGCTGATGTGTGTGGTTTTGAGAT				
Prsc9	KC753218	F: TCAGACTCCCGTGAACCAG	(tg)9	59.0	112-122	-
		R: CGCACTCGAGCAGCATCT				
Prsc10	KC753219	F: AACGCAACGGCCGCAACTAT	(ca)7(ct)7	56.5	260-284	-
		R: ACAAAACGCTCGAATACTGGGGG				
Prsc11	KC753220	F: CCCGGCAACTCAAATCAACTTCATA	(ac)11	62.7	229-371	244^b^
		R: GGTCTAATTCTATTGGTGGGCTCTCTGG				
Prsc12	KC753221	F: GGGGTGCATGTTGGGGATTG	(gt)10	59.0	185-223	220^b^
		R: TTTGGCCGGATTAAAACAGAGCA				
Prsc13	KC753222	F: CTTCACCATCACCGATTTCCCTT	(ctt)5	62.7	102	116
		R: GCAACGAAGCAGCTGAAGAACAC				

T_a_ - Optimal annealing temperature defined after gradient tests of the corresponding markers. ^a^Range of the fragment sizes from the polymorphic markers and the sequenced size of the monomorphic markers; ^b^Polymorphism observed for the transferred markers based on the 5 samples used.

The number of *P. rubriflora* alleles in the sampled remnants ranged from 3 to 12; the polymorphism information content (PIC) values of these markers ranged from 0.073 to 0.791, the observed heterozygosity (H_o_) ranged from 0.000 to 0.850, and the expected heterozygosity (H_e_) ranged from 0.000 to 0.835. No evidence of null alleles was observed, and no departure from Hardy-Weinberg equilibrium was observed (Table 2). No significant linkage disequilibrium (LD) was observed for any of the markers of this species after Bonferroni correction (*P*-value for 5% = 0.001389). The number of *P. ruscifolia* alleles in both of the remnants ranged from 3 to 17, the PIC values ranged from 0.289 to 0.883, the H_o_ values ranged from 0.040 to 0.783, and the H_e_ values ranged from 0.275 to 0.884. Possible null alleles were observed for the markers Prsc5, Prsc8 and Prsc9 from one remnant (ECD), and the markers Prsc4 and Prsc10 had possible null alleles in both remnants. A departure from HW was observed for Prsc2, Prsc5, Prsc6, Prsc8, Prsc9 and Prsc11 in one of the remnants (the majority were observed in ECD) and for Prsc4, Prsc7 and Prsc10 in both remnants (Table 3). Significant LD was observed for the loci Prsc5 and Prsc6 after Bonferroni correction (*P*-value for 1% = 0.00016).

**Table 2:** **Markers developed for *Prosopis rubriflora***

**Marker**	**N**_**a**_			**H**_**o**_		**H**_**e**_		**PIC**	**Null alleles**		**HW ( *P *-value)**	
	**T**	**FSM**	**FSV**	**FSM**	**FSV**	**FSM**	**FSV**		**FSM**	**FSV**	**FSM**	**FSV**
Prb1	7	6	7	0.526^a^	0.474	0.522^a^	0.737	0.602	0.044	0.145	0.348	0.045
Prb2	3	1	3	0.000	0.150^a^	0.000	0.145^a^	0.073	0.001	0.000	-	1.000
Prb3	3	3	2	0.316	0.150	0.428	0.296	0.313	0.103	0.134	0.101	0.069
Prb4	12	11	8	0.650	0.850^a^	0.799	0.803^a^	0.766	0.092	0.000	0.457	0.291
Prb5	4	4	4	0.500	0.600	0.583	0.683	0.576	0.039	0.007	0.689	0.254
Prb6	6	4	6	0.500^a^	0.400	0.458^a^	0.432	0.413	0.000	0.000	1.000	0.335
Prb7	4	4	4	0.684^a^	0.650^a^	0.605^a^	0.499^a^	0.473	0.000	0.000	0.355	0.121
Prb8	5	5	3	0.500	0.250	0.524	0.304	0.370	0.000	0.050	0.821	0.468
Prb9	10	10	9	0.684	0.650	0.835	0.819	0.791	0.053	0.062	0.177	0.016

FSM - Fazenda São Manoel, FSV - Fazenda Santa Vergínia, N_a_ - Number of alleles, H_o_ - Observed heterozygosity, H_e_ - Expected heterozygosity, PIC - Polymorphism information content, *P*-values of Hardy-Weinberg (HW) equilibrium (*P*-value > 0.0055 after Bonferroni correction), null alleles (null frequency < 0.20). ^a^Populations where the values of H_o_ were higher than those of H_e_.

**Table 3:** **Markers developed for *Prosopis ruscifolia***

**Marker**	**N**_**a**_			**H**_**o**_		**H**_**e**_		**PIC**	**Null alleles**		**HW ( *P *-value)**	
	**T**	**FRC**	**ECD**	**FRC**	**ECD**	**FRC**	**ECD**		**FRC**	**ECD**	**FRC**	**ECD**
Prsc1	7	4	7	0.600^a^	0.783	0.566^a^	0.828	0.701	0.000	0.020	0.916	0.009
Prsc2	8	7	5	0.480	0.565	0.553	0.761	0.658	0.032	0.114	0.034	0.00^c^
Prsc3	3	3	3	0.360^a^	0.348	0.344^a^	0.456	0.348	0.000	0.048	0.674	0.073
Prsc4	5	4	3	0.040	0.043	0.321	0.275	0.289	0.247^b^	0.221^b^	0.000^c^	0.000^c^
Prsc5	6	4	5	0.440^a^	0.273	0.378^a^	0.654	0.484	0.000	0.218^b^	1.000	0,000^c^
Prsc6	8	4	8	0.320	0.696	0.653	0.801	0.703	0.196	0.068	0.001^c^	0.087
Prsc7	9	6	7	0.440	0.565	0.727	0.779	0.755	0.167	0.081	0.001^c^	0.000^c^
Prsc8	7	4	7	0.320	0.174	0.577	0.789	0.656	0.165	0.338^b^	0.008	0.000^c^
Prsc9	5	3	3	0.240	0.130	0.280	0.559	0.430	0.040	0.267^b^	0.484	0.000^c^
Prsc10	7	5	5	0.286	0.227	0.633	0.758	0.670	0.212^b^	0.294^b^	0.000^c^	0.000^c^
Prsc11	17	8	12	0.560	0.652	0.845	0.884	0.883	0.157	0.127	0.003	0.000^c^
Prsc12	5	4	5	0.600	0.591	0.569	0.707	0.589	0.000	0.075	0.592	0.013

FRC - Fazenda Retiro Conceição, ECD - Estação do Carandazal, N_a_ - Number of alleles, H_o_ - Observed heterozygosity, H_e_ - Expected heterozygosity, PIC - Polymorphism information content, *P*-values of Hardy-Weinberg (HW) equilibrium (*P*-value > 0.0041 after Bonferroni correction). ^a^Populations where the values of H_o_ were higher than those of H_e_; ^b^Possible null alleles (null frequency < 0.20); ^c^Departure from HW equilibrium was observed.

Higher values of H_o_ were observed for the Prb1, Prb2, Prb4, Prb6, Prb7, Prsc1, Prsc3 and Prsc5 markers in this study; these higher values may indicate that an insufficient number of samples was collected or may be related to the reproductive patterns of these populations. The ECD populations are highly disturbed, and the FRC population is currently recovering from a relatively recent suppression (within the last 15 years); these factors may underlie the observed departure from HW and the presence of null alleles. A study with new and conserved populations may produce better results for these markers.

These markers are the first microsatellite markers developed for *Prosopis rubriflora* and *Prosopis ruscifolia,* and together with the set of *P. ruscifolia* markers amplified by Bessega *et al.*[20], they are expected to be useful tools for studies of the conservation genetics, reproductive biology, phylogeography and taxonomy of these species.

### Availability of supporting data

The original sequences of the developed markers were submitted to the GenBank database (http://ncbi.nlm.nih.gov), and the registered codes are available in Table 1.

The testimony samples were deposited at Herbarium Universidade Estadual de Campinas (UEC – Campinas, SP, BR) and registered according to the following: *P. rubriflora* – 74477 (Fazenda São Manoel – Porto Murtinho, MS), 154715 (Fazenda Santa Vergínia – Porto Murtinho, MS); *P. ruscifolia* – 74469 (Fazenda Retiro Conceição – Porto Murtinho, MS), 37266 (Estação do Carandazal – Corumbá, MS).

## Competing interests

The authors declare that no competing interests exist regarding this research.

## Authors’ contributions

FMA developed and characterized the markers and drafted the manuscript; MIZ provided support during the statistical analysis; ALBS provided support for the plant collection in the field; AMGAT participated in the experimental design; APS conceived the study, participated in its design and coordination and helped draft the manuscript. All of the authors read and approved the final manuscript.
